# Risk of Macrovascular and Microvascular Disease in Diabetes Diagnosed Using Oral Glucose Tolerance Test With and Without Confirmation by Hemoglobin A1c: The Whitehall II Cohort Study

**DOI:** 10.1161/CIRCULATIONAHA.122.059430

**Published:** 2022-08-25

**Authors:** Adam G. Tabák, Eric J. Brunner, Joni V. Lindbohm, Archana Singh-Manoux, Martin J. Shipley, Naveed Sattar, Mika Kivimäki

**Affiliations:** Department of Epidemiology and Public Health, University College London, UK (A.G.T., E.J.B., J.V.L., A.S.-M., M.J.S., M.K.).; Department of Internal Medicine and Oncology and Department of Public Health, Semmelweis University Faculty of Medicine, Budapest, Hungary (A.G.T.).; Clinicum, Faculty of Medicine, University of Helsinki, Finland (J.V.L., M.K.).; Université de Paris, Inserm U1153, Epidemiology of Ageing & Neurodegenerative Diseases, Paris, France (A.S.-M.).; BHF Glasgow Cardiovascular Research Centre, University of Glasgow, UK (N.S.).

**Keywords:** cardiovascular diseases, cohort studies, diabetes mellitus, type 2, glucose tolerance test, glycated hemoglobin A, renal insufficiency, chronic

## Abstract

It is unclear whether replacing oral glucose tolerance test (OGTT) with hemoglobin A1c (HbA1c) measurement for diagnosing diabetes is justified. We aimed to assess the proportion of OGTT-diagnosed diabetes cases that can be confirmed by HbA1c and to examine whether individuals with OGTT diagnosis but nondiagnostic HbA1c are at higher risk of macrovascular and microvascular disease.

**Methods:**

Participants were 5773 men and women from the population-based Whitehall II prospective cohort study in the United Kingdom. New OGTT diabetes cases diagnosed in clinical examinations in 2002 to 2004 and 2007 to 2009 were assessed for HbA1c confirmation (≥6.5%) in these and subsequent clinical examinations in 2012 to 2013 and 2015 to 2016. All participants were followed up for major cardiovascular events through linkage to electronic health records until 2017 and for incident chronic kidney disease (estimated glomerular filtration rate <60 mL·min^−1^·1.73 m^−2^) until the last clinical examination. In analysis of vascular disease risk, new OGTT-diagnosed diabetes cases with and without diagnostic HbA1c and preexisting diabetes cases were compared with diabetes-free participants.

**Results:**

Of the 378 (59.3%) participants with OGTT-diagnosed diabetes, 224 were confirmed by HbA1c during 4.1 years (SD, 4.1 years) of follow-up. We recorded 942 cardiovascular events over 12.1 years. After adjustment for nonmodifiable risk factors and compared with the 4997 diabetes-free participants, 371 participants with new HbA1c-confirmed diabetes and 405 participants with preexisting diabetes had increased risk of cardiovascular disease (hazard ratio, 1.53 [95% CI, 1.12–2.10] and 1.85 [95% CI, 1.50–2.28], respectively). The corresponding hazard ratios in the analysis of incident chronic kidney disease (487 cases; follow-up, 6.6 years) were 1.69 (95% CI, 1.09–2.62) for 282 participants with new HbA1c-confirmed diabetes and 1.67 (95% CI, 1.22–2.28) for 276 participants with preexisting diabetes. In both analyses, OGTT cases with nondiagnostic HbA1c (n=149 and 107) had a risk (hazard ratio, 0.99–1.07) similar to that of the diabetes-free population.

**Conclusions:**

More than 40% of OGTT-diagnosed diabetes cases were not confirmed by HbA1c during an extended follow-up. However, because these individuals have a risk of cardiovascular disease and chronic kidney disease similar to that of the diabetes-free population, replacement of OGTT with HbA1c-based diagnosis appears justified.

**Clinical Perspective**

**What Is New?**

- In a population-based cohort study with 5-year repeated oral glucose tolerance test (OGTT) and hemoglobin A1c (HbA1c) measurements, only 59.3% of OGTT-diagnosed diabetes cases were confirmed by HbA1c at the same or a subsequent examination during a 4.1-year follow-up.
- Incident OGTT-diagnosed diabetes cases with HbA1c confirmation and preexisting diabetes cases had similarly increased risk of cardiovascular disease (hazard ratio, 1.53 and 1.85) and chronic kidney disease (hazard ratio, 1.69 and 1.67), whereas unconfirmed OGTT cases had a risk similar to that of the diabetes-free population.

**What Are the Clinical Implications?**

- Because people with OGTT-diagnosed diabetes without diagnostic HbA1c have a risk of cardiovascular disease and chronic kidney disease similar to that of the diabetes-free population, replacement of OGTT with HbA1c-based diagnosis appears justified.
- There seems to be no need to consider OGTT when HbA1c and fasting glucose levels are apparently inconclusive; fasting glucose tests are needed only in exceptional circumstances in which HbA1c results are likely to be unreliable.
- These findings lend confidence to the widespread use of HbA1c for diagnosing diabetes in vast majority of clinical settings.

After hemoglobin A1c (HbA1c) was recommended by the International Expert Committee for the diagnosis of diabetes mellitus in 2009,^1^ several studies have highlighted the fact that the overlap between cases diagnosed by the gold standard oral glucose tolerance test (OGTT) and by HbA1c is limited.^2–6^ The sensitivity of HbA1c for detecting OGTT-diagnosed diabetes has ranged widely across populations (17%–78%),^2–6^ although it has been hypothesized that the overlap between OGTT-based and HbA1c-based diagnosis would increase over time. This diagnostic variation and concern not to increase diabetes prevalence substantially underpin recommendations to confirm diabetes status by repeating the same measurement on a different occasion.^1,7^

Unlike OGTT, measurement of HbA1c does not require fasting. For this and other reasons, HbA1c has largely replaced OGTT in diabetes screening and diagnosis.^7^ Few studies have repeat assessments of diabetes status, but based on a single measurement, high levels of both OGTT and HbA1c are associated with increased risk of microvascular disorders such as retinopathy, neuropathy, and diabetic kidney disease.^8–10^ With respect to macrovascular outcomes, some studies suggest that HbA1c predicts cardiovascular disease (CVD) events even after adjustment for fasting or postload blood glucose.^11–13^ However, none of these studies directly answer the pragmatic question of whether dropping OGTT from the diagnostic repertoire would mean that clinicians lose sight of a group of people with an elevated microvascular and macrovascular risk.

To address uncertainties about the consequences of moving from an OGTT/glucose-based diagnosis to an HbA1c-based diagnosis of diabetes, we set out to investigate the extent to which OGTT-diagnosed diabetes cases would be confirmed by HbA1c over time and whether the risk of CVD and incident chronic kidney disease (CKD) is elevated in people with OGTT-diagnosed diabetes if their HbA1c values remain in the nondiabetes range.

## Methods

### Setting and Study Design

The Whitehall II study is an occupational cohort study that was initiated in 1985 and recruited 10 308 participants (3413 women) who were 35 to 55 years from 20 London-based Civil Service departments.^14^ The baseline visit (phase 1) included a clinical examination and a self-administered questionnaire in 1985 to 1988 (response rate, 73%). During follow-up, repeated clinical examinations (phases 3, 5, 7, 9, 11, and 12 in 1991–1994, 1997–1999, 2002–2004, 2007–2009, 2012–2013, and 2015–2016) and additional postal questionnaire-only phases (phases 2, 4, 6, and 8 in 1988–1990, 1995–1996, 2001, and 2006) were performed. All participants without known diabetes had a standardized 75-g OGTT at clinic phases 3 to 9, and all participants had an HbA1c measurement at phases 7 to 12. This study was approved by University College London Hospital Committee on the Ethics of Human Research (reference 85/0938). Written informed consent from participants and research ethics approvals were renewed at each contact.

**Figure 1.**
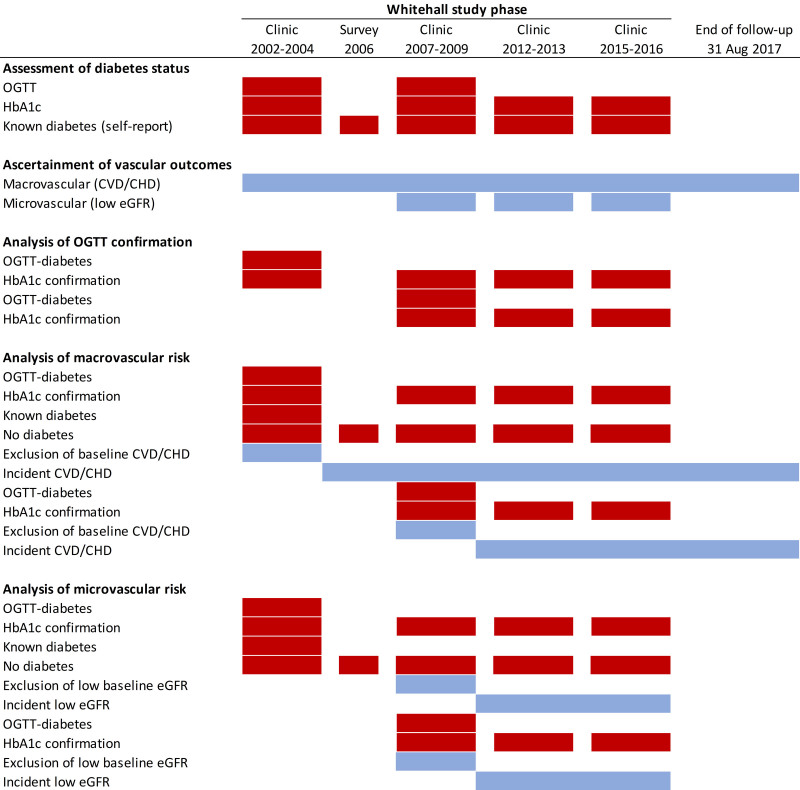
**Study design including study-related procedures and follow-ups for different analyses.** In analyses of macrovascular and microvascular risk, no diabetes refers to participants without diabetes at baseline and follow-up; known diabetes refers to diabetes cases who had diabetes diagnosed before attending baseline clinical examination; confirmed diabetes refers to oral glucose tolerance test (OGTT)–diagnosed diabetes in study clinic that was confirmed by hemoglobin A1c (HbA1c) test in the same or subsequent clinical examination; and unconfirmed diabetes refers to OGTT-diagnosed diabetes with normal HbA1c at baseline and follow-up. CHD indicates coronary heart disease; CVD, cardiovascular disease; and eGFR, estimated glomerular filtration rate.

Study designs for the analyses are described in detail in the Supplemental Methods and Figure 1. In brief, for confirmation of incident OGTT-diagnosed diabetes cases by HbA1c, we followed incident OGTT-based diabetes cases diagnosed at phases 7 or 9 until confirmation of diabetes status by HbA1c, self-report of doctor diagnosis, or treatment with antidiabetic medication. For analysis of CVD and CKD risk by diabetes status, in addition to the above incident diabetes cases, we followed up participants with preexisting diabetes diagnosed before phase 7 and those without diabetes diagnosis throughout the study. Follow-up for CVD (the macrovascular outcome) through linked electronic records was until August 2017. We defined CKD (the microvascular outcome) as estimated glomerular filtration rate (eGFR) <60 mL·min^−1^·1.73 m^−2^ and extended follow-up through phases 11 and 12.

### Data, Materials, and Code Disclosure Statement

Data, protocols, and other metadata of the Whitehall II study are available to the scientific community through either the Whitehall II study data–sharing portal^15^ or the Dementias Platform UK.^16^ The data that support the currently reported findings of this study are available from the corresponding author on reasonable request.

### Study Participants

**Figure 2.**
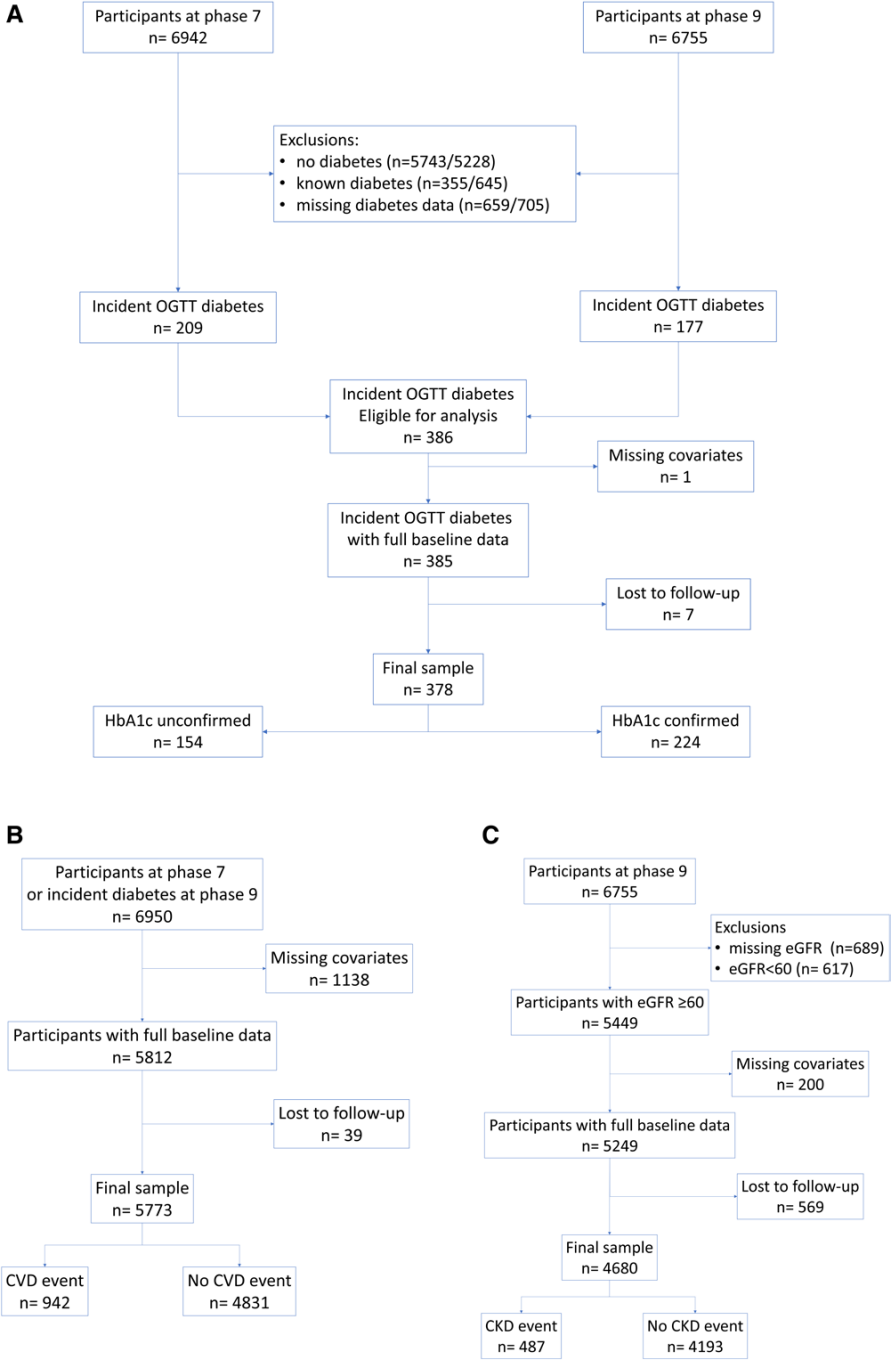
**Flowchart for selection of participants for analyses of HbA1c confirmation (A), macrovascular disease (B), and microvascular disease (C).** Covariates for analyses of hemoglobin A1c (HbA1c) confirmation (**A**): ethnicity, body mass index, high-density lipoprotein cholesterol (HDL-C), and HbA1c. Covariates for analyses of macrovascular and microvascular disease (**B** and **C**): ethnicity, social position, smoking, total cholesterol, HDL-C, systolic blood pressure, antihypertensive and lipid-lowering medication, and diabetes status. CKD indicates chronic kidney disease; CVD, cardiovascular disease; eGFR; estimated glomerular filtration rate; and OGTT, oral glucose tolerance test.

Figure 2 shows a flowchart for the selection of participants for analyses of HbA1c confirmation, CVD, and CKD. For confirmation of incident OGTT-diagnosed cases by HbA1c, we included 378 of 386 (97.9%) individuals and followed them up for 4.1±4.1 years (mean±SD). For analysis of CVD, we included 5773 of the 6950 (83.1%) participants (known diabetes, n=405; incident diabetes, n=371; no diabetes, n=4997) and followed them up for 12.1±3.3 years. For the analysis of CKD, we included 4680 of 5449 (85.9%) participants (known diabetes, n=276; incident diabetes, n=282; no diabetes, n=4122) and followed them up for 6.6±1.7 years.

### Measurement of Diabetes-Related Traits and Baseline Characteristics

Fasting and 2-hour postload venous blood samples were taken during a 75-g OGTT according to standardized protocols, and blood glucose was measured with the glucose oxidase method. HbA1c was measured on Tosoh G series analyzers using high-performance liquid chromatography. Results are reported as percentages according to National Glycohemoglobin Standardization Program recommendation.^17^

Known diabetes at phases 7 and 9 was defined as fasting glucose ≥7.0 mmol/L (to convert to milligrams per deciliter, multiply by 18) or a 2-hour postload glucose ≥11.1 mmol/L during OGTT at phase 3, 5, or 7 (the last for phase 9 diabetes only) or the use of antidiabetic medication or report of doctor-diagnosed diabetes at any screening or questionnaire phases before phase 7 or 9 (phase 9 diabetes only).^18^

Incident OGTT-based diabetes at baseline was diagnosed if fasting glucose, postload glucose, or both were above the cutoff at phase 7 or 9 and the participant had no known diabetes at phase 7 or 9.

HbA1c-confirmed diabetes was defined if an incident OGTT-diagnosed diabetes case had an HbA1c value ≥6.5% at the time of or after the diagnosis by OGTT (81.7% of all cases) or reported the use of antidiabetic medication or doctor-diagnosed diabetes after the diagnosis by OGTT (18.3%). The date of diabetes diagnosis was the date of the blood draw for cases diagnosed by HbA1c in the clinical examination and the midpoint between the last visit before diagnosis and the visit when the diagnosis outside the Whitehall clinic was first reported.^1,7,19^

The following baseline covariates were drawn from questionnaires or were measured during screening: age; sex; ethnicity (Office for National Statistics 1991 census types); occupational position (high: senior administrators; intermediate: executives, professionals, and technical staff; low: clerical and office support staff); smoking (current/past/never); weight; height; systolic and diastolic blood pressures; total cholesterol; triglycerides; high-density lipoprotein cholesterol (HDL-C); and 10-year CVD and coronary heart disease (CHD) risk (Framingham risk equation). Details are given in the Supplemental Methods.^20^

Prevalent CVD, defined as a CHD or stroke event that had occurred before baseline examination, was ascertained through data linkage to records from the NHS Hospital Episode Statistics database and the Office for National Statistics registry.^21^

### Ascertainment of Macrovascular and Microvascular Outcomes

Ascertainment of CVD events and their dates was based on data linkage to records from hospitalizations through the NHS Hospital Episode Statistics database for nonfatal CVD as a primary or secondary diagnosis and to the Office for National Statistics death registry by use of the NHS identification number. The main outcome for CVD was the first incident or recurrent CVD event (main analysis) and the first incident or recurrent CHD or incident CHD/CVD event (sensitivity analyses) after baseline.^21^

Serum creatinine was measured with a kinetic colorimetric (Jaffe) method.^22^ eGFR was calculated with the CKD Epidemiology Collaboration creatinine equation based on sex, age, race, and serum creatinine.^23^

Incident CKD was defined using data from a minimum of 2 repeated measurements of eGFR assuming a linear decline in eGFR with age.^24,25^ Individuals with <2 eGFR measurements and a baseline eGFR <60 mL·min^−1^·1.73 m^−2^ were excluded. We determined the slope (change in eGFR) and intercept (eGFR at baseline) for each participant using least-squares regression based on 2 to 3 eGFR measurements during follow-up. From the slope and intercept, we determined the level of eGFR at the end of follow-up. Incident CKD was indicated if an eGFR <60 mL·min^−1^·1.73 m^−2^ was reached during follow-up. For sensitivity analysis, we defined CKD as an estimated eGFR <45 mL·min^−1^·1.73 m^−2^ or an actual eGFR<60 mL·min^−1^·1.73 m^−2^ at phase 11 or 12 in participants with a baseline value ≥60 mL·min^−1^·1.73 m^−2^ (based on either estimation or actual value, respectively). Date of CKD diagnosis was defined as the estimated date to a value <45 mL·min^−1^·1.73 m^−2^ or the time elapsed between baseline and the abnormal eGFR measurement.

### Statistical Analysis

To investigate factors associated with the confirmation of OGTT-based incident diabetes by subsequent HbA1c measurement among participants with OGTT-diagnosed diabetes, we used Cox proportional hazard regression models with HbA1c confirmation (yes versus no) as the outcome, a positive OGTT diagnosis as the predictor, and time from baseline to confirmation or censoring as the underlying time. Hazard ratios (HRs) were adjusted for age, sex, ethnicity, body mass index, and HDL-C followed by fasting glucose at baseline. HRs for continuous variables are provided both as per 1 unit and as per 1-SD increment. To be able to include participants diagnosed by OGTT and HbA1c at the same time, follow-up time was set at 1 day for these cases. For graphical representation, unadjusted Kaplan-Meier survival curves were plotted.

For the analysis of factors associated with macrovascular and microvascular diseases, we applied Cox proportional hazard regression models with CVD or CKD as the outcome, diabetes status (no diabetes, incident OGTT based diabetes, known diabetes) as the predictor at baseline, confirmation status by HbA1c as a time-varying covariate, and time from baseline to diagnosis of CVD/CKD or censoring as the underlying time. This means that all follow-up until censoring or HbA1c assessment is counted for the OGTT diagnosis without confirmation group, and the follow-up after confirmation (or the whole follow-up time if the diagnosis is confirmed at the time of OGTT) is counted for the OGTT diagnosis confirmed by HbA1c group. HRs were first adjusted for age, sex, ethnicity, occupational position, and prevalent CVD (model 1), followed by modifiable cardiovascular risk factors (smoking, total cholesterol, HDL-C, systolic blood pressure, and the use of lipid- and blood pressure–lowering medication; model 2). We estimated excess risk mediated by modifiable risk factors by calculating percentage attenuation in model 1 β coefficient after inclusion of risk factors in model 2:

Percentage of excess risk mediated by risk factors=100×(β_Model 1_−β_Model 2_)/(β_Model 1_).

We calculated the 95% CI around the percentage attenuation by using a bootstrap method with 1000 resamplings in Stata (version 15.1). For all Cox models, the proportionality assumption was tested visually with Schoenfeld residuals.

To assess whether we accrued enough events for analyses of CVD and CKD, we performed post hoc power calculations based on the cumulative incidence of events in the nondiabetic population and the number of OGTT-based diabetes cases with and without HbA1c confirmation.^26^ Given a cumulative incidence of 14.8% for CVD and 9.7% for CKD over the follow-up in the control group and the number of participants in the nondiabetic (n=4997 in CVD and n=4129 in CKD analysis), confirmed diabetes (n=222 and n=175), and unconfirmed diabetes (n=149 and n=107) groups, we have 80% power with an α of 0.05 to detect an HR of 1.5 among confirmed cases and 1.6 among unconfirmed cases for CVD and 1.7 for confirmed and 1.9 for unconfirmed cases for CKD.

To investigate the robustness of our results, several sensitivity analyses were performed that are reported in the Supplemental Material (Table S2 and Figures S1–S3). First, we investigated the association between diabetes status at baseline and follow-up and alternative outcomes (new CHD, incident CHD, incident CVD for macrovascular disease; estimated eGFR <45 mL·min^−1^·1.73 m^−2^, measured eGFR <60 mL·min^−1^·1.73 m^−2^ for CKD). Second, we excluded diseases that could potentially affect the validity of HbA1c measurements (ie, meaningfully alter HbA1c levels independently of glycemia per se) and diseases that could lead to increased vascular risk independently of glycemia. Thus, we excluded participants with self-reported anemia (CVD/CKD, n=78/71; *International Classification of Diseases, 10th Revision* codes D46*, D5*, and D61*–64*) and cases of systemic autoimmune diseases (CVD/CKD, n=164/138; codes M05*–M09*, M30*–M36*) at baseline or during follow-up for these analyses. Third, to eliminate potential effect of baseline examinations spanning over phases 7 and 9 of the study, we ran an analysis including only incident OGTT cases diagnosed at phase 7.

Two-sided *P* values were used with an α level of 0.05 for statistical significance. All statistical analyses were performed with SPSS version 24 for Windows.

### Patient and Public Involvement

Participants of the Whitehall II study and members of the public were not involved in setting the research question or the outcome measures, nor were they involved in developing plans for recruitment, design, or implementation of the study. We recognize the importance of public involvement in instigating change in policy and practice, but funding for these activities was not available. All results are disseminated to study participants via newsletters and our website, which has a participant portal,^27^ and to a larger audience through media outreach.

## Results

The HbA1c values of OGTT-diagnosed cases ranged from as low as 4.1% to 12.8% (mean, 6.4%), with a substantial proportion of HbA1c values below the diagnostic cutoff for diabetes (Figure S1).

Cases with OGTT-diagnosed confirmed by HbA1c were older and less frequently of White ethnicity; had a lower occupational position and higher body mass index, fasting and 2-hour glucose, and HbA1c; and were more frequently diagnosed on the basis of both high fasting and 2-hour glucose values. They also presented with a lower HDL-C level, a higher triglyceride level, and a higher estimated 10-year cardiovascular risk (all *P*<0.05). No differences in the sex distribution, total cholesterol, estimated GFR, use of blood pressure– and lipid-lowering medications, or prevalent CVD were observed (all *P*>0.15; Table 1).

**Table 1.**
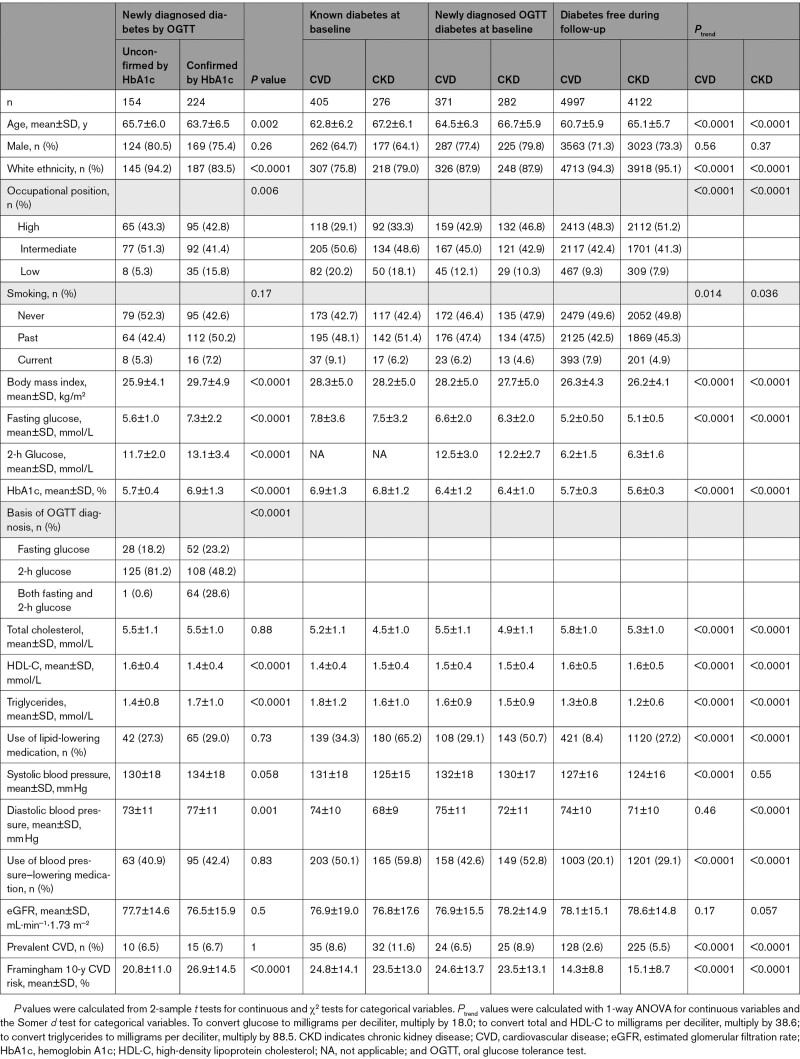
Baseline Characteristics of Participants With OGTT-Diagnosed Diabetes at Baseline by Confirmation Status Based on Hba1c and Those Followed-Up for CVD and CKD Events by Diabetes Status at Baseline

**Figure 3.**
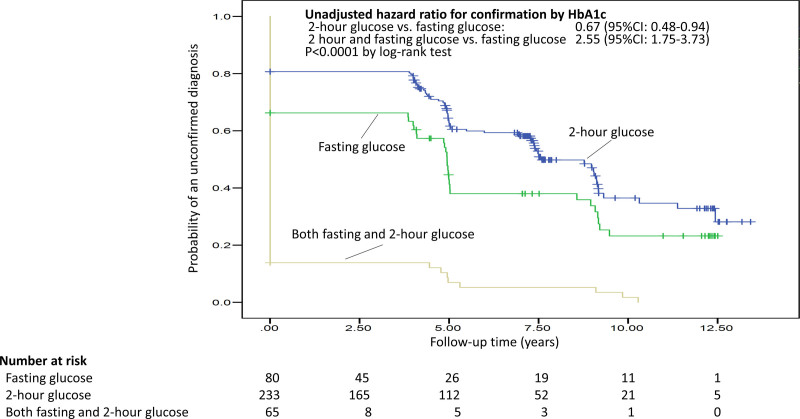
**Kaplan-Meier estimates for HbA1c confirmation of OGTT-diagnosed diabetes by type of abnormal glucose value (fasting vs 2-hour vs both).** HbA1c indicates hemoglobin A1c.

Of the 378 participants with incident OGTT-based diabetes at phase 7 or 9, 160 (42.3%) had their diabetes diagnosis confirmed by HbA1c at the same phase and 224 (59.3%) at a subsequent phase. All but 1 participant (n=64 of 65) with both an abnormal fasting and 2-hour glucose value had their diagnosis confirmed by HbA1c during follow-up (HR for confirmation by HbA1c, 2.55 [95% CI, 1.17–3.73]). In contrast, only half of the 313 participants with either high fasting or high 2-hour glucose (but not both) met HbA1c-based diagnosis after 5.6 years (95% CI, 4.4–6.7) and 7.7 years (95% CI, 6.9–8.4) of follow-up, respectively (Figure 3). When glycemia-related variables (even including fasting glucose) were controlled for, the number of abnormal glucose values remained a predictor of diabetes confirmation by HbA1c during follow-up (HR for confirmation by HbA1c, 2.00 [95% CI, 1.34–2.97]; Table 2 and Table S1).

**Table 2.**
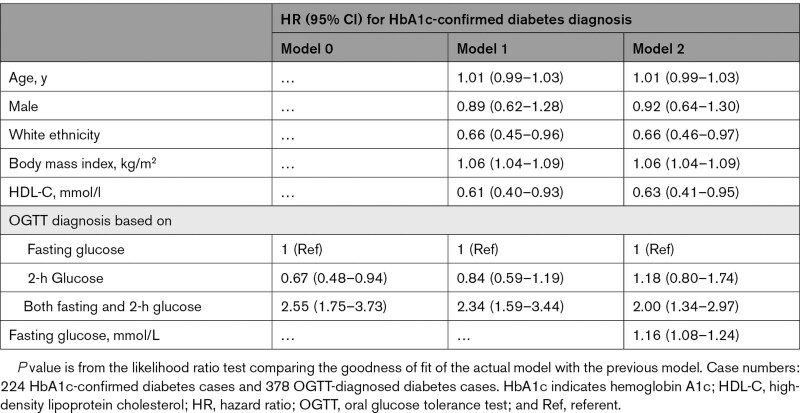
Cox Proportional Hazard Models for Confirmation of OGTT-Based Diabetes Diagnosis by HbA1c Test During Follow-Up

We observed an increasing trend from nondiabetic through incident diabetes to known diabetes cases in regard of age; proportion of non-White ethnicity, lower occupational position, and current or past smoking; body mass index; fasting glucose; HbA1c; triglycerides; use of lipid- and blood pressure–lowering medication; frequency of prevalent CVD; and 10-year CVD risk. We also found an inverse trend for total cholesterol, HDL-C, and systolic blood pressure in the CVD/CHD population and diastolic blood pressure across these groups for the CKD population. No clear association with sex or eGFR was observed (Table 1).

**Figure 4.**
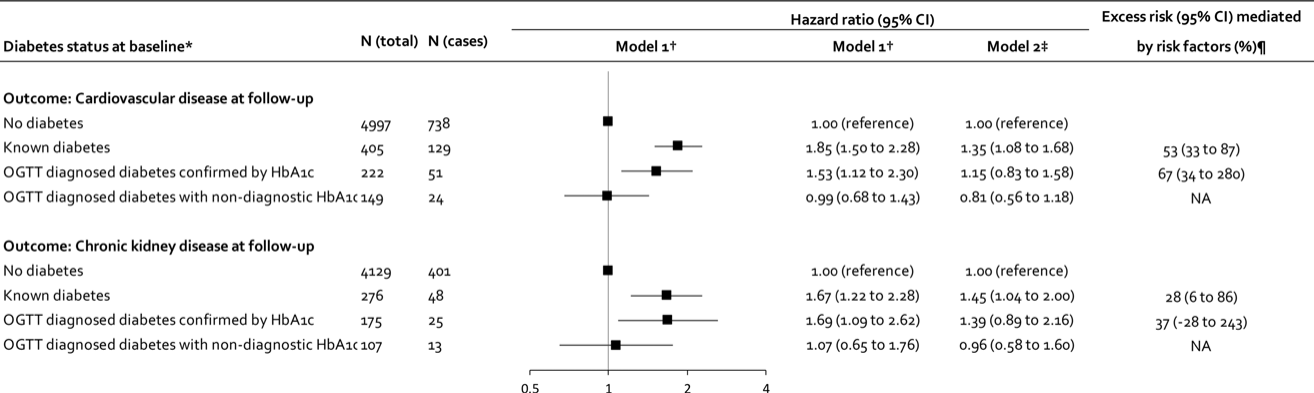
**HRs for new-onset CVD and incident CKD according to diabetes status at baseline.** *No diabetes refers to participants without diabetes at baseline and follow-up. Known diabetes refers to diabetes cases who had diabetes diagnosed before attending baseline clinical examination. Oral glucose tolerance test (OGTT)–diagnosed diabetes refers to OGTT-diagnosed diabetes at study clinic either confirmed or not confirmed by hemoglobin A1C test in the same or subsequent clinical examination. †Model 1: adjusted for age, sex, ethnicity, occupational position, and prevalent cardiovascular disease (CVD) at baseline. ‡Model 2: as model 1 but additionally adjusted for smoking, total cholesterol, high-density lipoprotein cholesterol, systolic blood pressure, and use of antihypertensive and lipid-lowering medication. ¶Excess risk mediated by modifiable risk factors listed in Model 2. CKD indicates chronic kidney disease; HR, hazard ratio; and NA, not applicable.

Because of the important differences in baseline vascular factors between diabetes groups, we investigated the association of diabetes status with risk of vascular complications in 2 nested multivariable-adjusted models (Figure 4). The first model, adjusted for nonmodifiable risk factors, showed a 1.67- and 1.85-fold increased risk of CVD and CKD for participants with known diabetes, a 1.53 and 1.69-fold increased risk for those with HbA1c-confirmed OGTT-diagnosed diabetes (all *P*<0.05), but no increased risk in participants with OGTT-diagnosed diabetes not confirmed by HbA1c. After further adjustment for modifiable vascular risk factors, a substantial attenuation of the HRs for both known diabetes and HbA1c-confirmed incident diabetes was observed, suggesting that a significant part of the excess risk was attributable to modifiable vascular risk factors.

Sensitivity analyses with new and incident CHD and incident CVD and CKD events as the outcomes largely confirmed the main analysis by showing a 1.9 to 2.1 times increased risk in people with known diabetes and a 1.4 to 3.1 times increased risk in people with HbA1c-confirmed diabetes in models adjusted for nonmodifiable vascular risk factors, whereas unconfirmed OGTT-based cases had a risk similar to that in the nondiabetic population (Figure S1). The exclusion of self-reported anemia or systemic autoimmune diseases had no clear effect on the risk associated with diabetes status (Figure S2). The exclusion of incident OGTT diabetes cases at phase 9 limited statistical power but had no effect on point estimates (Table S2 and Figure S3).

## Discussion

In the British Whitehall II cohort study, we confirmed the limited cross-sectional overlap between OGTT-based and HbA1c-based diagnoses and found that this remained the case during a mean follow-up of 4.1 years. Only half of people with either abnormal fasting or 2-hour glucose value in OGTT but practically all with both values abnormal met HbA1c criteria for diabetes during follow-up. People with an HbA1c-confirmed diabetes diagnosis had an increased risk of CVD and incident CKD, whereas OGTT-only diabetes cases had a disease risk similar to that of those without diabetes in analyses adjusted for age, sex, ethnicity, and social position.

### Strengths and Weaknesses of the Study

Our study has limitations. First, OGTT and the measurement of HbA1c were not performed at entry to the Whitehall II study. Thus, the baseline for the current analyses was at subsequent clinical examinations when, inevitably, some sample attrition had already occurred. Furthermore, the diagnosis of diabetes was based on only 1 OGTT or HbA1c test, whereas guidelines recommend repeating the abnormal test before diagnosis.^1,7^ Only ≈70% of diabetes cases from a single test can be confirmed by a second fasting or postload glucose,^28^ but the much lower coefficient of variation for HbA1c implies that this percentage is higher for a single HbA1c test.^28^ Second, the participants were from an occupational cohort, with two-third of participants being men. It is unclear whether our findings are generalizable to the general population, ethnic minority groups, and women. However, although the healthy worker effect could bias absolute risks, validation studies suggest that relative risks are similar to those found in population-based studies.^29^ CVD outcomes were ascertained with national registry linkage to hospitalization records, which may be prone to ascertainment and other types of bias. There is little reason, however, to assume that ascertainment would be different for people with and without confirmed diabetes. We estimated the date of CKD diagnosis on the unproven assumption that there is linear decrease in eGFR over time.^24,25^ However, sensitivity analyses based on actual measures of eGFR replicated the main findings. Because no eGFR measurement was done at phase 7 of the study, we had a shorter follow-up time for the CKD analysis, leading to wider CIs around the estimates.

The strengths of our study include the use of gold standard methods for OGTT and HbA1c measurements. Blood draws and OGTTs were performed according to standardized operating procedures to minimize both preanalytical and analytical variation, and all HbA1c measurements were standardized to the Diabetes Control and Complications Trial method.^17^ The robustness of our findings is further supported by the unequivocal results of our main and several sensitivity analyses. This study may be the largest study available with repeated and simultaneous measures of both OGTT and HbA1c levels and vascular outcomes with a relatively long follow-up. The frequency of blood draws (3–5 years) is similar to that recommended by clinical guidelines for average-risk people, making our results directly relevant for clinical practice.^1,7,30^ Given the decreasing use of OGTT in clinical practice and research studies (except for the diagnosis of gestational diabetes), few future studies will have adequate data to address these clinically relevant questions, including those related to microvascular complications.

### Strengths and Weaknesses in Relation to Other Studies

Although there is a substantial body of research on cross-sectional overlap between diabetes diagnostic methods, surprisingly little evidence is available on the convergence of these methods over time.^6,31–33^ If baseline OGTT is treated as the test under investigation and HbA1c as the gold standard, then the rate at which incident diagnosis is confirmed reflects the positive predictive value of the baseline test; it was only 59% over the mean 4-year follow-up. This agrees with the findings from the Atherosclerosis Risk in Communities study.^33^

Given the uncertainty of the diagnosis based on a single abnormal value, it has been suggested that diabetes is overdiagnosed and thus the frequency of undiagnosed diabetes may actually be lower than previously thought.^31,34^ Because of its much lower coefficient of variation, the use of HbA1c could potentially reduce the rate of false positives.^17,28^ Given the strong association between HbA1c levels and the risk of clinically diagnosed diabetes, our findings support the hypothesis that during an extended follow-up, most high-risk people will be diagnosed with diabetes using HbA1c.^32^

Several studies of macrovascular disease suggest that high fasting glucose, postload glucose, and HbA1c are all associated with increased risk of CVD and CHD events,^11–13,35–37^ but in direct comparisons, HbA1c appears to be the strongest predictor.^11,35,36^ In addition, there are at least 2 studies in which HbA1c remained a predictor of macrovascular events after adjustment for glucose values.^12,13^ These observations are consistent with our finding suggesting increased risk of macrovascular complications for OGTT-diagnosed diabetes in the presence but not absence of elevated HbA1c values.

Evidence suggests that all glycemic measures (fasting and postload glucose and HbA1c) mark an increased risk of diabetic retinopathy and nephropathy.^8–10^ However, it is not clear which of the measures is the strongest predictor and whether there is a threshold on the scale of glycemia that is predictive of these complications.^8–11,37^ Our findings favor HbA1c in analysis of 1 microvascular end point, CKD, the most commonly assessed microvascular event.

The International Expert Committee has based its criteria of diabetes diagnosis on the risk of long-term complications such as diabetic retinopathy.^1^ OGTT used to be the gold standard for diagnosis, but our findings, in combination with the above-described equivocal evidence, suggest that OGTT is not superior to HbA1c. Given the emphasis of current guidelines on cardiometabolic screening, HbA1c rather than OGTT will be the dominant test for diabetes in the future. It is important to clarify whether high-risk people are lost with this shift in diagnostic criteria. Our study suggests that the OGTT-positive–only group has a risk of major CVD and CKD similar to that of the nondiabetic population. This should be reassuring to clinicians using HbA1c for diabetes diagnosis.

### Conclusions

These findings suggest that pickup of CVD and CKD risk is not harmed but rather improved by the preferential use of HbA1c for the diagnosis of diabetes. Therefore, there appears no need to consider OGTT when HbA1c levels or fasting glucose levels are inconclusive, as stated in recent guidelines.^30^ Fasting glucose tests are needed only in exceptional circumstances when HbA1c results are likely to be unreliable.^1,17^

## Article Information

### Acknowledgments

The authors thank all of the participating civil service departments and their welfare, personnel, and establishment officers; the British Occupational Health and Safety Agency; the British Council of Civil Service Unions; all participating civil servants in the Whitehall II study; and all members of the Whitehall II study team. The Whitehall II study team comprises research scientists, statisticians, study coordinators, nurses, data managers, administrative assistants, and data entry staff, who make the study possible. A.G.T. and M.K. developed the hypothesis and study design. A.G.T. did the statistical analysis. A.G.T. and M.K. wrote the first and successive drafts of the manuscript. All authors conceived and designed the study, analyzed and interpreted the data, and drafted or critically revised the manuscript for important intellectual content or, in addition, acquired data. E.J.B., A.S.-M., and M.K. obtained funding for the Whitehall II study. A.G.T. and M.K. are the guarantors. A.G.T. and M.K. had full access to the data and take responsibility for the integrity of the data and the accuracy of the data analysis. The corresponding author attests that all listed authors meet authorship criteria and that no others meeting the criteria have been omitted.

### Sources of Funding

The Whitehall II study is supported by grants from the Wellcome Trust (221854/Z/20/Z), the Medical Research Council (K013351, R024227), the British Heart Foundation (RG/13/2/30098), and the National Institute on Aging (National Institutes of Health, R01AG056477, R01AG062553). The funders had no role in considering the study design or in the collection, analysis, interpretation of data; writing of the report; or decision to submit the article for publication.

### Disclosures

Dr Tabák was supported by the UK Medical Research Council (S011676), NordForsk (the Nordic Research Programme on Health and Welfare, 75021), and the Ministry of Innovation and Technology of Hungary from the National Research, Development and Innovation Fund (2021 Thematic Excellence Programme funding scheme, TKP2021-NKTA-47). Prof Kivimäki was supported by the Wellcome Trust (221854/Z/20/Z), the Medical Research Council (K013351, R024227, S011676), the National Institute on Aging (National Institutes of Health, R01AG056477, R01AG062553), and NordForsk (75021). Prof Sattar is supported by the British Heart Foundation Centre of Research Excellence Grant (RE/18/6/34217). Prof Sattar has consulted for Amgen, AstraZeneca, Boehringer Ingelheim, Eli-Lilly, Hanmi Pharmaceuticals, Novo Nordisk, Novartis, Roche Diagnostics, Sanofi, and Pfizer. Prof Sattar has also received grant funding, paid to his university, from AstraZeneca, Boehringer Ingelheim, Novartis, and Roche Diagnostics. The other authors report no conflicts.

### Supplemental Material

Supplemental Methods

Tables S1 and S2

Figures S1–S4

## Supplementary Material



## References

[1] International Expert Committee. International Expert Committee report on the role of the A1C assay in the diagnosis of diabetes. Diabetes Care. 2009;32:1327–1334. 19502545 10.2337/dc09-9033 PMC2699715

[2] van ‘t Riet E Alssema M Rijkelijkhuizen JM Kostense PJ Nijpels G Dekker JM. Relationship between A1C and glucose levels in the general Dutch population: the New Hoorn study. Diabetes Care. 2010;33:61–66. doi: 10.2337/dc09-0677 19808928 10.2337/dc09-0677 PMC2797987

[3] Christensen DL Witte DR Kaduka L Jorgensen ME Borch-Johnsen K Mohan V Shaw JE Tabak AG Vistisen D. Moving to an A1C-based diagnosis of diabetes has a different impact on prevalence in different ethnic groups. Diabetes Care. 2010;33:580–582. doi: 10.2337/dc09-1843 20009099 10.2337/dc09-1843 PMC2827511

[4] Barry E Roberts S Oke J Vijayaraghavan S Normansell R Greenhalgh T. Efficacy and effectiveness of screen and treat policies in prevention of type 2 diabetes: systematic review and meta-analysis of screening tests and interventions. BMJ. 2017;356:i6538. doi: 10.1136/bmj.i6538 28052845 10.1136/bmj.i6538

[5] NCD Risk Factor Collaboration (NCD-RisC). Effects of diabetes definition on global surveillance of diabetes prevalence and diagnosis: a pooled analysis of 96 population-based studies with 331,288 participants. Lancet Diabetes Endocrinol. 2015;3:624–637. doi: 10.1016/S2213-8587(15)00129-1 26109024 10.1016/S2213-8587(15)00129-1 PMC4673089

[6] Warren B Pankow JS Matsushita K Punjabi NM Daya NR Grams M Woodward M Selvin E. Comparative prognostic performance of definitions of prediabetes: a prospective cohort analysis of the Atherosclerosis Risk in Communities (ARIC) study. Lancet Diabetes Endocrinol. 2017;5:34–42. doi: 10.1016/S2213-8587(16)30321-7 27863979 10.1016/S2213-8587(16)30321-7 PMC5183486

[7] American Diabetes Association. 2. Classification and diagnosis of diabetes: standards of medical care in diabetes–2019. Diabetes Care. 2019;42:S13–S28. doi: 10.2337/dc19-S002 30559228 10.2337/dc19-S002

[8] Sabanayagam C Liew G Tai ES Shankar A Lim SC Subramaniam T Wong TY. Relationship between glycated haemoglobin and microvascular complications: is there a natural cut-off point for the diagnosis of diabetes? Diabetologia. 2009;52:1279–1289. doi: 10.1007/s00125-009-1360-5 19387611 10.1007/s00125-009-1360-5

[9] Wong TY Liew G Tapp RJ Schmidt MI Wang JJ Mitchell P Klein R Klein BEK Zimmet P Shaw J. Relation between fasting glucose and retinopathy for diagnosis of diabetes: three population-based cross-sectional studies. Lancet. 2008;371:736–743. doi: 10.1016/S0140-6736(08)60343-8 18313502 10.1016/S0140-6736(08)60343-8 PMC2350208

[10] Cheng YJ Gregg EW Geiss LS Imperatore G Williams DE Zhang X Albright AL Cowie CC Klein R Saaddine JB. Association of A1C and fasting plasma glucose levels with diabetic retinopathy prevalence in the U.S. population: implications for diabetes diagnostic thresholds. Diabetes Care. 2009;32:2027–2032. doi: 10.2337/dc09-0440 19875604 10.2337/dc09-0440 PMC2768189

[11] Metcalf PA Kyle C Kenealy T Jackson RT. HbA1c in relation to incident diabetes and diabetes-related complications in non-diabetic adults at baseline. J Diabetes Complications. 2017;31:814–823. doi: 10.1016/j.jdiacomp.2017.02.007 28319002 10.1016/j.jdiacomp.2017.02.007

[12] Selvin E Steffes MW Zhu H Matsushita K Wagenknecht L Pankow J Coresh J Brancati FL. Glycated hemoglobin, diabetes, and cardiovascular risk in nondiabetic adults. N Engl J Med. 2010;362:800–811. doi: 10.1056/NEJMoa0908359 20200384 10.1056/NEJMoa0908359 PMC2872990

[13] Park S Barrett-Connor E Wingard DL Shan J Edelstein S. GHb is a better predictor at cardiovascular disease than fasting or postchallenge plasma glucose in women without diabetes: the Rancho Bernardo Study. Diabetes Care. 1996;19:450–456. doi: 10.2337/diacare.19.5.450 8732708 10.2337/diacare.19.5.450

[14] Marmot M Brunner E. Cohort profile: the Whitehall II study. Int J Epidemiol. 2005;34:251–256. doi: 10.1093/ije/dyh372 15576467 10.1093/ije/dyh372

[15] UCL. Whitehall II study data sharing. Accessed July 13, 2022. https://www.ucl.ac.uk/epidemiology-health-care/research/epidemiology-and-public-health/research/whitehall-ii/data-sharing

[16] Dementias Platform UK. Welcome to Dementias Platform UK. Accessed July 13, 2022. https://www.dementiasplatform.uk/

[17] Sacks DB; ADA/EASD/IDF Working Group of the HbA1c Assay. Global harmonization of hemoglobin A1c. Clin Chem. 2005;51:681–683. doi: 10.1373/clinchem.2004.047431 15788784 10.1373/clinchem.2004.047431

[18] Tabak AG Jokela M Akbaraly TN Brunner EJ Kivimaki M Witte DR. Trajectories of glycaemia, insulin sensitivity, and insulin secretion before diagnosis of type 2 diabetes: an analysis from the Whitehall II study. Lancet. 2009;373:2215–2221. doi: 10.1016/S0140-6736(09)60619-X 19515410 10.1016/S0140-6736(09)60619-X PMC2726723

[19] Use of Glycated Haemoglobin (HbA1c) in the Diagnosis of Diabetes Mellitus: Abbreviated Report of a WHO Consultation. World Health Organization; 2011. 26158184

[20] Bouillon K Batty GD Hamer M Sabia S Shipley MJ Britton A Singh-Manoux A Kivimaki M. Cardiovascular disease risk scores in identifying future frailty: the Whitehall II prospective cohort study. Heart. 2013;99:737–742. doi: 10.1136/heartjnl-2012-302922 23503403 10.1136/heartjnl-2012-302922 PMC3632981

[21] Kivimaki M Batty GD Singh-Manoux A Britton A Brunner EJ Shipley MJ. Validity of cardiovascular disease event ascertainment using linkage to UK hospital records. Epidemiology. 2017;28:735–739. doi: 10.1097/EDE.0000000000000688 28570383 10.1097/EDE.0000000000000688 PMC5540351

[22] Al-Qaoud TM Nitsch D Wells J Witte DR Brunner EJ. Socioeconomic status and reduced kidney function in the Whitehall II study: role of obesity and metabolic syndrome. Am J Kidney Dis. 2011;58:389–397. doi: 10.1053/j.ajkd.2011.04.017 21719176 10.1053/j.ajkd.2011.04.017 PMC3192873

[23] Inker LA Schmid CH Tighiouart H Eckfeldt JH Feldman HI Greene T Kusek JW Manzi J Van Lente F Zhang YL; CKD-EPI Investigators. Estimating glomerular filtration rate from serum creatinine and cystatin C. N Engl J Med. 2012;367:20–29. doi: 10.1056/NEJMoa1114248 22762315 10.1056/NEJMoa1114248 PMC4398023

[24] Derose SF Rutkowski MP Crooks PW Shi JM Wang JQ Kalantar-Zadeh K Kovesdy CP Levin NW Jacobsen SJ. Racial differences in estimated GFR decline, ESRD, and mortality in an integrated health system. Am J Kidney Dis. 2013;62:236–244. doi: 10.1053/j.ajkd.2013.01.019 23499049 10.1053/j.ajkd.2013.01.019 PMC3723721

[25] Eriksen BO Ingebretsen OC. The progression of chronic kidney disease: a 10-year population-based study of the effects of gender and age. Kidney Int. 2006;69:375–382. doi: 10.1038/sj.ki.5000058 16408129 10.1038/sj.ki.5000058

[26] Rothman KJ Greenland S Lash TL. Modern Epidemiology. 3rd ed. Wolters Kluwer Health/Lippincott Williams & Wilkins; 2008.

[27] UCL. Participants’ area. Accessed July 13, 2022. https://www.ucl.ac.uk/epidemiology-health-care/research/epidemiology-and-public-health/research/whitehall-ii/participants-area

[28] Selvin E Crainiceanu CM Brancati FL Coresh J. Short-term variability in measures of glycemia and implications for the classification of diabetes. Arch Intern Med. 2007;167:1545–1551. doi: 10.1001/archinte.167.14.1545 17646610 10.1001/archinte.167.14.1545

[29] Batty GD Shipley M Tabak A Singh-Manoux A Brunner E Britton A Kivimaki M. Generalizability of occupational cohort study findings. Epidemiology. 2014;25:932–933. doi: 10.1097/EDE.0000000000000184 25265141 10.1097/EDE.0000000000000184

[30] Cosentino F Grant PJ Aboyans V Bailey CJ Ceriello A Delgado V Federici M Filippatos G Grobbee DE Hansen TB; ESC Scientific Document Group. 2019 ESC guidelines on diabetes, pre-diabetes, and cardiovascular diseases developed in collaboration with the EASD. Eur Heart J. 2020;41:255–323. doi: 10.1093/eurheartj/ehz486 31497854 10.1093/eurheartj/ehz486

[31] Selvin E Wang D Lee AK Bergenstal RM Coresh J. Identifying trends in undiagnosed diabetes in U.S. adults by using a confirmatory definition: a cross-sectional study. Ann Intern Med. 2017;167:769–776. doi: 10.7326/M17-1272 29059691 10.7326/M17-1272 PMC5744859

[32] Zhang X Gregg EW Williamson DF Barker LE Thomas W Bullard KM Imperatore G Williams DE Albright AL. A1C level and future risk of diabetes: a systematic review. Diabetes Care. 2010;33:1665–1673. doi: 10.2337/dc09-1939 20587727 10.2337/dc09-1939 PMC2890379

[33] Selvin E Wang D Matsushita K Grams ME Coresh J. Prognostic implications of single-sample confirmatory testing for undiagnosed diabetes: a prospective cohort study. Ann Intern Med. 2018;169:156–164. doi: 10.7326/M18-0091 29913486 10.7326/M18-0091 PMC6082697

[34] Geiss LS Bullard KM Brinks R Gregg EW. Considerations in epidemiologic definitions of undiagnosed diabetes. Diabetes Care. 2018;41:1835–1838. doi: 10.2337/dc17-1838 30135196 10.2337/dc17-1838

[35] Eastwood SV Tillin T Mayet J Shibata DK Wright A Heasman J Beauchamp N Forouhi NG Hughes AD Chaturvedi N. Ethnic differences in cross-sectional associations between impaired glucose regulation, identified by oral glucose tolerance test or HbA1c values, and cardiovascular disease in a cohort of European and South Asian origin. Diabet Med. 2016;33:340–347. doi: 10.1111/dme.12895 26314829 10.1111/dme.12895 PMC4740925

[36] Eastwood SV Tillin T Sattar N Forouhi NG Hughes AD Chaturvedi N. Associations between prediabetes, by three different diagnostic criteria, and incident CVD differ in South Asians and Europeans. Diabetes Care. 2015;38:2325–2332. doi: 10.2337/dc15-1078 26486189 10.2337/dc15-1078 PMC4868252

[37] Xing FY Neeland IJ Gore MO Ayers CR Paixao AR Turer AT Berry JD Khera A de Lemos JA McGuire DK. Association of prediabetes by fasting glucose and/or haemoglobin A1c levels with subclinical atherosclerosis and impaired renal function: observations from the Dallas Heart Study. Diab Vasc Dis Res. 2014;11:11–18. doi: 10.1177/1479164113514239 24344119 10.1177/1479164113514239 PMC5728654

