# Supplementary material for: Risk of Macrovascular and Microvascular Disease in Diabetes Diagnosed Using Oral Glucose Tolerance Test With and Without Confirmation by Hemoglobin A1c: The Whitehall II Cohort Study
Source: Circulation. 2022 Aug 25;146(13):995–1005. doi: 10.1161/CIRCULATIONAHA.122.059430 (PMC9508989; doi:10.1161/CIRCULATIONAHA.122.059430)
Supplement: Supplementary file 1 [file cir-146-995-s001.pdf]

SUPPLEMENTAL MATERIAL

Risk of Macro- and Microvascular Disease in Diabetes Diagnosed Using Oral Glucose Tolerance Test with and without Confirmation by Haemoglobin A1c: the Whitehall II Cohort Study

Adam G. Tabák, MD, PhD[1,2,3], Eric J. Brunner, PhD, FFPH [1], Joni V. Lindbohm, MD PhD[1,4], Archana Singh-Manoux, PhD[1,5], Martin J. Shipley, MSc[1], Naveed Sattar, FMedSci [6], Mika Kivimäki, FMedSci [1,4]

[1] Department of Epidemiology and Public Health, University College London, London, UK

[2] Department of Internal Medicine and Oncology, Semmelweis University Faculty of Medicine, Budapest, Hungary

[3] Department of Public Health, Semmelweis University Faculty of Medicine, Budapest, Hungary

[4] Clinicum, Faculty of Medicine, University of Helsinki, Helsinki, Finland

[5] Université de Paris, Inserm U1153, Epidemiology of Ageing & Neurodegenerative diseases, Paris, France

[6] BHF Glasgow Cardiovascular Research Centre, University of Glasgow, Glasgow, UK

Table of contents

Supplemental Methods 3

Study participants and design 3

Measurement of diabetes related traits and baseline characteristics 4

Ascertainment of macrovascular and microvascular outcomes 5

Supplemental Tables 7

Table S1. Cox proportional hazard models for confirmation of OGTT-based diabetes diagnosis by HbA1c test during follow-up 7

Table S2. Cox proportional hazard models for confirmation of OGTT-based diabetes diagnosis at phase 7 by HbA1c test during follow-up 8

Supplemental Figures 9

Figure S1. Overlay scatterplot of HbA1c by fasting glucose (blue markers) and postload glucose (red markers) from 378 participants with OGTT-diagnosed incident diabetes at baseline 9

Figure S2. Hazard ratios for new and incident coronary heart disease (CHD), incident cardiovascular disease (CVD) and incident chronic kidney disease (two alternative definitions) according to diabetes status at baseline and follow-up 10

Figure S3. Hazard ratios for new-onset cardiovascular disease and incident chronic kidney disease according to diabetes status at baseline and follow-up after exclusion of participants with anaemia (n=78/71 in CVD/CKD analysis) or systemic autoimmune disease (n=164/138) 12

Figure S4. Hazard ratios for new-onset cardiovascular disease and incident chronic kidney disease according to diabetes status at phase 7 and follow-up 13

Supplemental Methods

Study participants and design

Figure 1 shows a flow chart for selection of participants for analyses of HbA1c confirmation, macrovascular disease and microvascular disease. For confirmation of incident OGTT-diagnosed cases by HbA1c, there were 386 participants with incident diabetes diagnosed at either phase 7 (n=209) or phase 9 (n=177). Of these, we excluded 8 due to missing covariates at baseline (ethnicity, BMI, HDL-cholesterol, HbA1c) or lost to follow-up, leaving 378 individuals (Phase 7 n=202, Phase 9 n= 176) for the analysis of confirmation of diabetes diagnosis by HbA1c (n=224 cases, follow-up [mean±SD] 4.1±4.1 years).

For analysis of macrovascular disease, there were a total of 6950 eligible participants, of whom we excluded 1138 individuals due to missing covariates at baseline (ethnicity, social position, smoking, HDL-c, LDL-c, systolic blood pressure, antihypertensive and lipid lowering medication, and diabetes status) and a further 39 individuals with no follow-up for CHD/CVD outcomes via linked electronic records from the Hospital Episode Statistics database, leaving 5773 participants (known diabetes n=405, incident diabetes n=371, no diabetes n=4997) for the analysis of CVD/CHD outcomes (n=942/788 events, follow-up 12.1±3.3 years).

For the analysis of microvascular disease, there were a total of 5449 participants with eGFR≥60 ml/min/1.73 m^2 at baseline. Of these, we excluded 200 individuals due to missing covariates at baseline (ethnicity, social position, smoking, HDL-c, LDL-c, systolic blood pressure, antihypertensive and lipid lowering medication) and 569 individuals due to lost to follow-up, leaving 4680 participants (known diabetes n=276, incident diabetes n=282, no diabetes n=4122) for the analysis of CKD (n=487 events, follow-up 6.6±1.7 years).

Overall study design is presented in **Figure 1**. For confirmation of incident OGTT-diabetes cases by HbA1c (the first analysis), we followed incident OGTT-based diabetes cases diagnosed at Phases 7 or 9 from diagnosis through Phases 7 to 12, until confirmation of diabetes status by HbA1c, self-report of doctor diagnosis, or treatment with antidiabetic medication.

For analysis of CVD and CKD risk by diabetes status (the second analysis), in addition to the above incident diabetes cases, we followed up participants with pre-existing diabetes diagnosed before Phase 7 and those without diabetes diagnosis throughout the study. For CVD (the macrovascular outcome), follow-up started at Phase 7 for participants with pre-

existing diabetes, those with incident diabetes diagnosed at Phase 7, and participants free of diabetes throughout the study. For participants with incident diabetes diagnosed at Phase 9, the follow-up started at Phase 9. All eligible participants (irrespective of previous CVD status) were followed up for new events of CVD via linked electronic records until August 2017. As kidney function (serum creatinine) was measured for the first time at Phase 9, this is the baseline for the CKD follow-up. We defined CKD as estimated glomerular filtration rate [eGFR]<60 ml/min/1.73 m2. Participants without CKD at baseline were followed up for incident CKD through Phases 11 and 12.

Measurement of diabetes related traits and baseline characteristics

Fasting and 2-hour postload venous blood samples were taken during a 75g OGTT according to standardized protocols (Phases 3, 5, 7, 9). *Blood glucose* was measured with the glucose oxidase method (YSI Corporation, Yellow Springs, OH, USA).[18]

HbA1c was measured on Tosoh G5 (Phase 7 - 2002), Tosoh G7 (Phases 7 – 2003-2004 and Phase 9), and on a Tosoh G8 (Phases 11 and 12) analysers using gold-standard high-performance liquid chromatography [HPLC] method. Results are reported as percentages according to National Glycohemoglobin Standardization Program [NGSP] recommendation.[17]

Age and *sex* were based on questionnaire data. *Ethnicity* was defined according to the Office for National Statistics 1991 census types. Self-reported ethnicity at Phase 5 was mainly used; missing data were complemented by observer-assigned ethnicity from Phase 1.[14] British civil service employment grade was used as a measure of *occupational position* and was grouped into three categories: high (senior administrators), intermediate (executives, professionals, and technical staff) and low (clerical and office support staff).

Smoking status at baseline was measured using standard questionnaires and was classified into 3 categories (current/past/never). If information on smoking was missing at Phase 9, it was imputed using Phase 7 data. *Weight* and *height* (in the Frankfort plane) were measured in light clothing on a Soehnle scale to the nearest 0.1 kg and to the nearest 0.1 cm. Body mass index (BMI) was calculated as weight (kg)/height^2 (m). *Systolic and diastolic blood pressure* was measured twice in the sitting position after 5 minutes of rest with an OMRON HEM 907 machine. The average of the 2 readings was used in analysis.

Total cholesterol and *triglycerides* were determined by an enzymatic colorimetric procedure on a Roche Integra system at Phase 7, a Roche P Modular system at Phase 9 and on a COBAS 8000 at Phases 11 and 12. High-density-lipoprotein (*HDL)-cholesterol* was measured by direct determination using a homogenous enzymatic colorimetric test on a Roche Integra system at Phase 7, a Roche P Modular system at Phase 9 and on a COBAS 8000 at Phases 11 and 12.[20]

The use of *blood pressure and lipid lowering medication* was assessed based on a questionnaire requesting the use of all doctor-prescribed medication within the last 14 days. Antihypertensive medications included diuretics, beta/blockers, ACE inhibitors, angiotensin receptor blockers, calcium channel blockers, and other blood pressure lowering medications. Lipid lowering medications included statins, fibrates, niacin, ezetimibe, and other lipid lowering medications.

To determine estimated CVD and CHD risk at baseline, we calculated the *10-year CVD and CHD risk* using the Framingham risk equations.[20]

Ascertainment of macrovascular and microvascular outcomes

Ascertainment of *CHD events* and their dates was based on data linkage to records from hospitalizations through HES database for nonfatal CHD as a primary or secondary diagnosis (defined using the International Statistical Classification of Diseases and Related Health Problems, version 9 [ICD-9] codes 410–414, ICD-10 codes I20–I25) and to records of coronary death (defined using ICD-9 codes 410–414 and ICD-10 codes I20–I25) through the ONS death registry by using the NHS identification number.[21]

CVD events were defined as CHD events and/or non-fatal and fatal stroke (defined using ICD-9 codes 430, 431, 434, 436 and ICD-10-cased I60, I61, I63, I64). Ascertainment was based on data linkage to the HES database and ONS death registry as described for CHD. The main outcome for CVD was the first incident or recurrent CVD event (main analysis) and the first incident or recurrent CHD, or incident CHD/CVD event (sensitivity analysis) after baseline.[21]

Serum *creatinine* was measured using a kinetic colorimetric (Jaffe) method on a Roche "P" Modular system (Phase 9) and on a COBAS 8000 system (Phase 11 and 12).[22]

We determined the slope (change in *eGFR*) and intercept (eGFR at baseline) for each participant with least-squares regression based on their 2 to 3 eGFR measurement during follow-up. using the following equation:

$Slope_{eGFR} = \sum((time_i - time_{mean}) * (eGFR_i - eGFR_{mean})/\sum((time_i - time_{mean})^2)$

$Intercept_{eGFR} = eGFR_{mean} - Slope_{eGFR} * time_{mean}$

Supplemental Tables

Table S1. Cox proportional hazard models for confirmation of OGTT-based diabetes diagnosis by HbA1c test during follow-up

	**Hazard ratio (95% confidence interval) for HbA1c confirmed diabetes diagnosis**		
	**Model 0**	**Model 1**	**Model 2**
Age, per SD	NA	1.07 (0.93-1.22)	1.06 (0.92-1.23)
Male (vs female)	NA	0.89 (0.62-1.28)	0.92 (0.64-1.30)
White ethnicity (vs non-White)	NA	0.66 (0.45-0.96)	0.66 (0.46-0.97)
Body mass index, per SD	NA	1.35 (1.19-1.53)	1.35 (1.19-1.54)
HDL-cholesterol, per SD	NA	0.82 (0.70-0.97)	0.83 (0.70-0.98)
OGTT diagnosis based on:			
(i) Fasting glucose	1 (ref)	1 (ref)	1 (ref)
(ii) 2-hour glucose	0.67 (0.48-0.94)	0.84 (0.59-1.19)	1.18 (0.80-1.74)
(iii) Both fasting and 2-hour glucose	2.55 (1.75-3.73)	2.34 (1.59-3.44)	2.00 (1.34-2.97)
Fasting glucose, per SD	NA	NA	1.32 (1.16-1.52)

Abbreviations. HR, hazard ratio; 95% CI, 95% confidence interval

SD for age is 6.1 years, BMI 4.9 kg/m^2, HDL 0.4 mmol/l, and fasting glucose 1.9 mmol/l.

Case numbers: 224 HbA1c confirmed diabetes cases and 378 OGTT-diagnosed diabetes cases.

Table S2. Cox proportional hazard models for confirmation of OGTT-based diabetes diagnosis at phase 7 by HbA1c test during follow-up

	**Hazard ratio (95% confidence interval) for HbA1c confirmed diabetes diagnosis**		
	**Model 0**	**Model 1**	**Model 2**
Age, years	NA	1.00 (0.97-1.03)	0.99 (0.96-1.02)
Male	NA	1.02 (0.65-1.61)	0.97 (0.61-1.52)
White ethnicity	NA	0.56 (0.35-0.88)	0.58 (0.37-0.91)
Body mass index, kg/m^2	NA	1.05 (1.01-1.08)	1.05 (1.01-1.08)
HDL-cholesterol, mmol/l	NA	0.66 (0.39-1.12)	0.68 (0.40-1.14)
OGTT diagnosis based on:-			
(i) Fasting glucose	1 (ref)	1 (ref)	1 (ref)
(ii) 2-hour glucose	0.80 (0.53-1.21)	1.03 (0.66-1.61)	1.40 (0.86-2.28)
(iii) Both fasting and 2-hour glucose	2.50 (1.60-3.91)	2.37 (1.50-3.76)	2.12 (1.32-3.39)
Fasting glucose, mmol/l	NA	NA	1.15 (1.06-1.24)

Abbreviations. HR, hazard ratio; 95% CI, 95% confidence interval.

Case numbers: 140 HbA1c-confirmed diabetes cases and 202 OGTT-diagnosed diabetes cases.

Supplemental Figures

Figure S1. Overlay scatterplot of HbA1c by fasting glucose (blue markers) and postload glucose (red markers) from 378 participants with OGTT-diagnosed incident diabetes at baseline

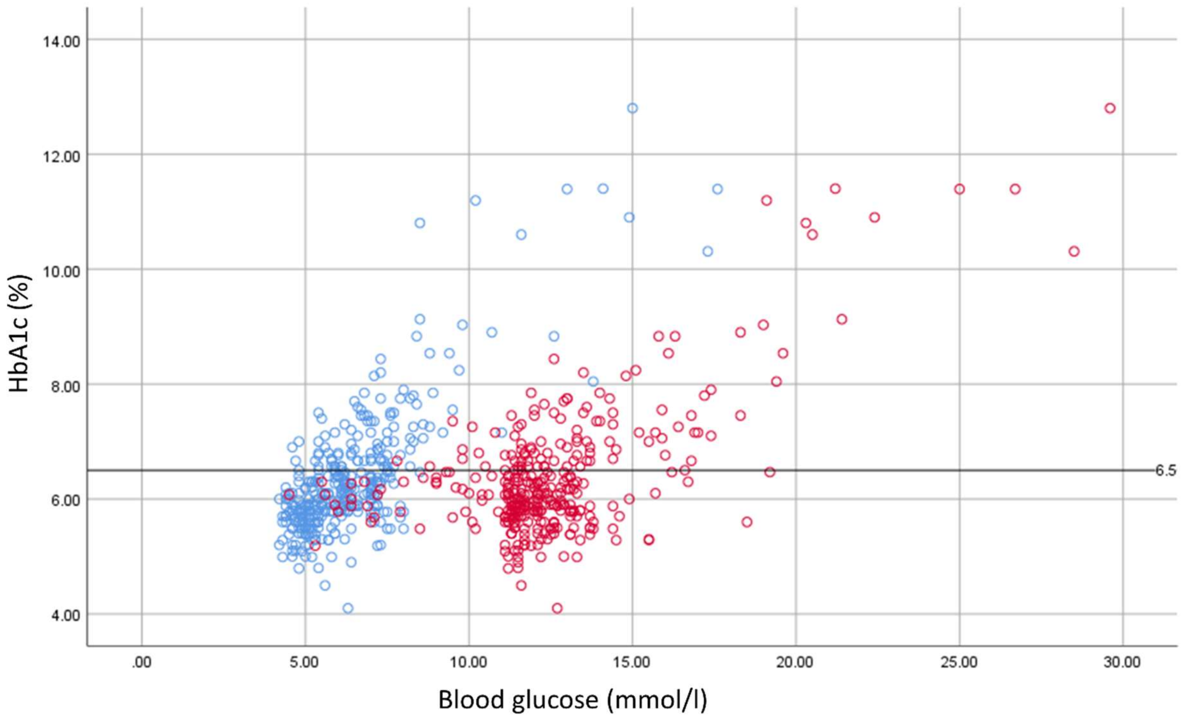

Figure S2. Hazard ratios for new and incident coronary heart disease (CHD), incident cardiovascular disease (CVD) and incident chronic kidney disease (two alternative definitions) according to diabetes status at baseline and follow-up

	N observations, N (cases)	Hazard ratio (95% CI)*	Hazard ratio (95% CI)*
**Outcome: New coronary heart disease**			
No diabetes	613, 4997		1.00 (reference)
Known diabetes	113, 405		1.9 (1.51 to 2.4)
Confirmed diabetes	40, 222		1.45 (1.02 to 2.06)
Unconfirmed diabetes	22, 149		1.01 (0.68 to 1.51)
**Outcome: Incident coronary heart disease**			
No diabetes	525, 4877		1.00 (reference)
Known diabetes	86, 373		1.94 (1.53 to 2.45)
Confirmed diabetes	30, 206		1.36 (0.91 to 2.02)
Unconfirmed diabetes	16, 141		0.89 (0.56 to 1.39)
**Outcome: Incident cardiovascular disease**			
No diabetes	645, 4869		1.00 (reference)
Known diabetes	102, 370		1.86 (1.5 to 2.31)
Confirmed diabetes	41, 206		1.5 (1.06 to 2.11)
Unconfirmed diabetes	18, 141		0.89 (0.59 to 1.34)
**Outcome: Incident chronic kidney disease (estimated eGFR<45 ml/min/m2**			
No diabetes	69, 4122		1.00 (reference)
Known diabetes	13, 276		2.05 (1.1 to 3.82)
Confirmed diabetes	10, 175		3.08 (1.45 to 6.55)
Unconfirmed diabetes	2, 107		1.41 (0.51 to 3.88)
**Outcome: Incident chronic kidney disease (eGFR<60 ml/min/m2)**			
No diabetes	358, 3819		1.00 (reference)
Known diabetes	44, 243		2 (1.45 to 2.76)
Confirmed diabetes	22, 158		1.78 (1.14 to 2.77)
Unconfirmed diabetes	16, 100		1.49 (0.9 to 2.48)

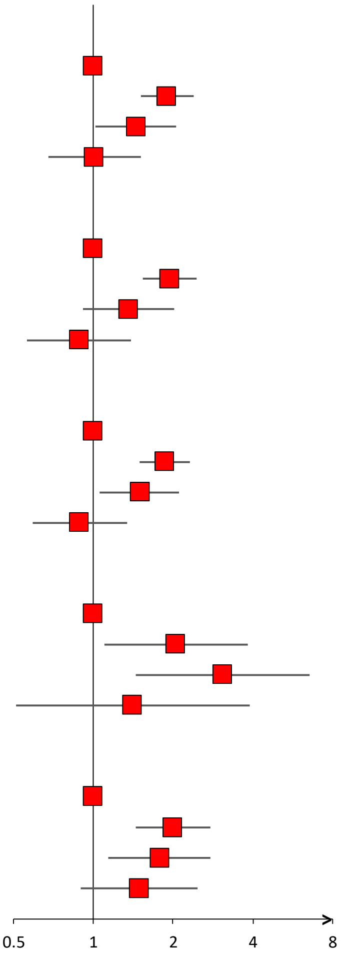

*Adjusted for age, sex, ethnicity, occupational position, and prevalent CVD (for new coronary heart disease and CKD outcomes)

No diabetes refers to participants without diabetes at baseline and follow-up.

Known diabetes refers to diabetes cases who had diabetes diagnosed before attending to baseline clinical examination.

Confirmed diabetes refers to OGTT-diagnosed diabetes in the study clinic which was confirmed by HbA1c test in the same or subsequent clinical examination.

Unconfirmed diabetes cases refer to OGTT-diagnosed diabetes with normal HbA1c values at baseline and follow-up.

Hazard ratios (HR) were estimated using Cox proportional hazards models adjusted for non-modifiable risk factors by diabetes status at baseline and during follow-up. HbA1c confirmation of incident OGTT diagnosed diabetes was treated as time-varying co-variant.

Figure S3. Hazard ratios for new-onset cardiovascular disease and incident chronic kidney disease according to diabetes status at baseline and follow-up after exclusion of participants with anaemia (n=78/71 in CVD/CKD analysis) or systemic autoimmune disease (n=164/138)

	N (cases), N (observations)	Hazard ratio (95% CI)*
**Outcome: Cardiovascular disease**		
No diabetes	708, 4902	1.00 (reference)
Known diabetes	121, 384	1.86 (1.5 to 2.31)
Confirmed diabetes	49, 218	1.5 (1.09 to 2.05)
Unconfirmed diabetes	23, 147	0.9 (0.63 to 1.33)
**Outcome: Chronic kidney disease**		
No diabetes	382, 4007	1.00 (reference)
Known diabetes	44, 258	1.68 (1.21 to 2.32)
Confirmed diabetes	24, 172	1.66 (1.06 to 2.61)
Unconfirmed diabetes	13, 105	1.1 (0.67 to 1.83)

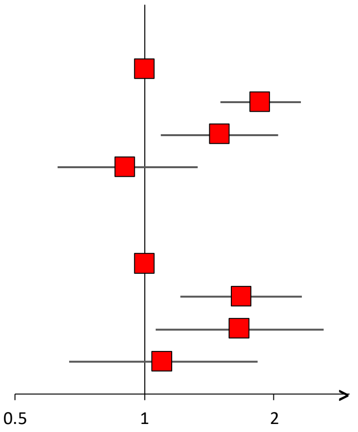

*Adjusted for age, sex, ethnicity, occupational position, and prevalent CVD

No diabetes refers to participants without diabetes at baseline and follow-up.

Known diabetes refers to diabetes cases who had diabetes diagnosed before attending to baseline clinical examination.

Confirmed diabetes refers to OGTT-diagnosed diabetes in study clinic which was confirmed by HbA1c test in the same or subsequent clinical examination.

Unconfirmed diabetes refers to OGTT-diagnosed diabetes with normal HbA1c at baseline and follow-up.

Hazard ratios (HR) were estimated using Cox proportional hazards models adjusted for non-modifiable risk factors by diabetes and prediabetes status at baseline and during follow-up. HbA1c confirmation of incident OGTT diagnosed diabetes/prediabetes was treated as time-varying covariate.

Figure S4. Hazard ratios for new-onset cardiovascular disease and incident chronic kidney disease according to diabetes status at phase 7 and follow-up

	N (cases), N (observations)	Hazard ratio (95% CI)*
**Outcome: Cardiovascular disease**		
No diabetes	736, 5037	1.00 (reference)
Known diabetes	129, 405	1.88 (1.52 to 2.32)
Confirmed diabetes	36, 141	1.73 (1.22 to 2.47)
Unconfirmed diabetes	13, 63	0.95 (0.57 to 1.6)
**Outcome: Chronic kidney disease**		
No diabetes	401, 4122	1.00 (reference)
Known diabetes	48, 276	1.65 (1.2 to 2.25)
Confirmed diabetes	20, 108	2.03 (1.26 to 3.26)
Unconfirmed diabetes	4, 36	0.87 (0.36 to 2.11)

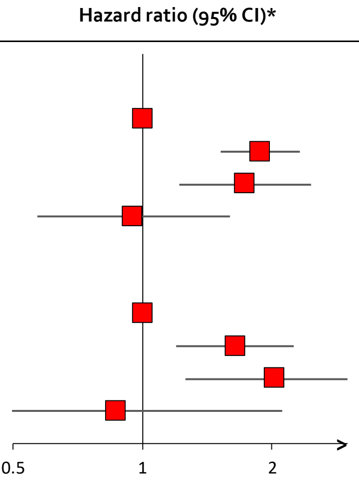

*Adjusted for age, sex, ethnicity, occupational position, and prevalent CVD

No diabetes refers to participants without diabetes at baseline and follow-up.

Known diabetes refers to diabetes cases who had diabetes diagnosed before attending to baseline clinical examination.

Confirmed diabetes refers to OGTT-diagnosed diabetes in study clinic which was confirmed by HbA1c test in the same or subsequent clinical examination.

Unconfirmed diabetes refers to OGTT-diagnosed diabetes with normal HbA1c at baseline and follow-up.

Hazard ratios (HR) were estimated using Cox proportional hazards models adjusted for non-modifiable risk factors by diabetes and prediabetes status at baseline and during follow-up. HbA1c confirmation of incident OGTT diagnosed diabetes/prediabetes was treated as time-varying covariate.
